# Reasons for ceiling ratings in real-life evaluations of hearing aids: the relationship between SNR and hearing aid ratings

**DOI:** 10.3389/fdgth.2023.1134490

**Published:** 2023-08-03

**Authors:** Nadja Schinkel-Bielefeld, Jana Ritslev, Dina Lelic

**Affiliations:** ^1^ R&D PSA SA DE ERL, WS Audiology, Erlangen, Germany; ^2^ Department of Nordic Studies and Linguistics, University of Copenhagen, Copenhagen, Denmark; ^3^ R&D PSA SA DE ERL, WS Audiology, Lynge, Denmark

**Keywords:** acceptable noise level, SNR, EMA, hearing aid evaluation, real-life hearing, adaptation, ecological momentary assessment

## Abstract

**Introduction:**

In past Ecological Momentary Assessment (EMA) studies, hearing aid outcome ratings have often been close to ceiling.

**Methods:**

To analyze the underlying reasons for the very positive ratings, we conducted a study with 17 experienced hearing aid wearers who were fitted with study hearing aids. The acceptable noise level and the noise level where participants were unable to follow speech were measured. The participants then rated hearing aid satisfaction, speech understanding and listening effort for pre-defined SNRs between −10 and +20 dB SPL in the laboratory. These ratings were compared to ratings of a two-week EMA trial. Additionally, estimates of SNRs were collected from hearing aids during the EMA trial and we assessed whether the participants experienced those SNRs rated poorly in the laboratory in real life.

**Results:**

The results showed that for hearing aid satisfaction and speech understanding, the full rating scale was used in the laboratory, while the ratings in real life were strongly skewed towards the positive end of the scale. In the laboratory, SNRs where participants indicated they could not follow the narrator (“unable to follow” noise level) were rated clearly better than the lowest possible ratings. This indicates that very negative ratings may not be applicable in real-life testing. The lower part of the distribution of real-life SNR estimates was related to participants’ individual “unable to follow” noise levels and the SNRs which were rated poorly in the laboratory made up less than 10% of the speech situations experienced in real life.

**Discussion:**

This indicates that people do not seem to frequently experience listening situations at SNRs where they are dissatisfied with their hearing aids and this could be the reason for the overly positive hearing aid outcome ratings in EMA studies. It remains unclear to what extent the scarcity of such situations is due lack of encounters or intentional avoidance.

## Introduction

Hearing aid outcomes can be affected by acoustic environments and listening demands that hearing aid users encounter in everyday life. There is a recent wave of Ecological Momentary Assessment (EMA) studies investigating these outcomes in real life [see Holube et al. (1) for an overview]. EMA is a “repeated sampling” questionnaire method that in hearing research is often used to ask participants to repeatedly report about their current listening environment. In contrast to retrospective questionnaires, EMA is not subject to memory bias and offers contextual information about the current listening situation. Jenstad et al. (2) showed that EMA responses provided more detail about individual variability across acoustic conditions than retrospective questions when looking at satisfaction, benefit, and residual activity limitation. Similarly, Andersson et al. (3) showed that real-life benefit from directional microphone and noise management processing in hearing aids was driven by preferences in acoustic environments classified as “speech” or “speech in noise”, which was not disentangled from the retrospective Speech, Spatial, and Qualities Hearing Scale questionnaire. A few other studies have used EMA to investigate real-life benefit of hearing aids and observed significant effects when contrasts were large. For example, Wu et al. (4) have shown that participants were more satisfied with hearing aids equipped with advanced directional microphone / noise reduction features, although there was no evidence supporting the benefit of premium noise reduction features or hearing aids over basic ones. Also, von Gablenz et al. (5) showed that hearing aid intervention led to better speech understanding as well as decreased listening effort and diminished disability—an effect that was pronounced in first-time hearing aid users, but very small in experienced hearing aid users looking to get a new hearing aid.

Although previous EMA studies have been able to show real-life benefits of hearing aid use, the ratings have generally been skewed toward the positive end of the scale (4). EMA studies looking into hearing aid satisfaction in everyday listening situations have observed a similar phenomenon (2, 6, 7). This led us to question, why do people tend to give such positive ratings? Is performance of modern hearing aids really that good or are there other reasons for high ratings seen in EMA studies? One proposed hypothesis is that people shape their acoustic environments to their needs and consequently improve or circumvent difficult listening situations. In many cases, listening conditions can be improved by easy measures such as turning up the TV volume, closing a window when it is noisy outside, moving closer to a conversation partner, or support hearing with lip reading. In an EMA study this could reduce the number of situations which may otherwise lead to lower hearing aid outcome ratings. Many measures to improve the listening situation also increase the SNR and previous studies have indicated that people spend most time in easy listening situations without much noise (4, 8, 9, 10). Another reason for high satisfaction / benefit ratings could be that EMA questionnaires do not capture difficult situations as participants are less inclined to interact with the study equipment and answer a survey in a situation that is already challenging for them (11). There could also be personal reasons for the high ratings, such as, wanting to please the experimenter in cases where a hearing aid / fitting is being evaluated by the hearing aid manufacturer or the participant's hearing care provider.

Not answering surveys in difficult situations can especially be problematic in cases where a new hearing aid feature that is expected to optimize these infrequently reported listening experiences is evaluated. EMA results that do not capture such situations could lead to wrong conclusions. Also, if two hearing aid programs are compared and difficult situations are modified or in the extreme case avoided due to dissatisfactory hearing aid performance for one program, but not the other, the EMA method could in turn have lower sensitivity to detect a difference between programs. This would be a problem for hearing aid manufacturers evaluating new features, but also when EMA is used in rehabilitation or in the process of choosing and fitting new hearing aids. Hence, understanding whether behavioral adaptations are the underlying reasons behind high hearing aid satisfaction ratings in EMA studies is relevant for development and rehabilitation alike. Collected objective hearing aid data can make such adaptation behavior discernible and might help to judge the suitability of a hearing program for a particular situation.

The overarching purpose of the current study was to investigate the reasons behind high hearing aid outcome ratings in EMA studies. To do this, we conducted a combined laboratory and EMA study. We assessed the acceptable noise levels (ANL) as well as hearing aid outcomes at different signal-to-noise ratios (SNRs) rated on EMA scales in the laboratory. We then compared how these laboratory ratings relate to real-life EMA ratings during a 17-day field trial. Our research questions (RQ) were: RQ1: what is the range of the scale ratings in real-life in comparison to the laboratory?; RQ2: how are speech situations at the ANL rated in EMA?; RQ3: what percentage of time during the field trial do participants spend in environments where SNRs are worse than their ANLs or in SNRs that have been rated as dissatisfactory in the laboratory?; and RQ4: are the ANLs measured in the laboratory and the SNRs experienced during the field trial related?

## Materials and methods

Ethical clearance for conducting the study was obtained from the Research Ethics Committee of the Capital Region of Denmark (case no. H-18056647). The participants received written and oral instructions about the aims of the current study. They then signed an informed consent form.

### Participants

**Figure 1:**
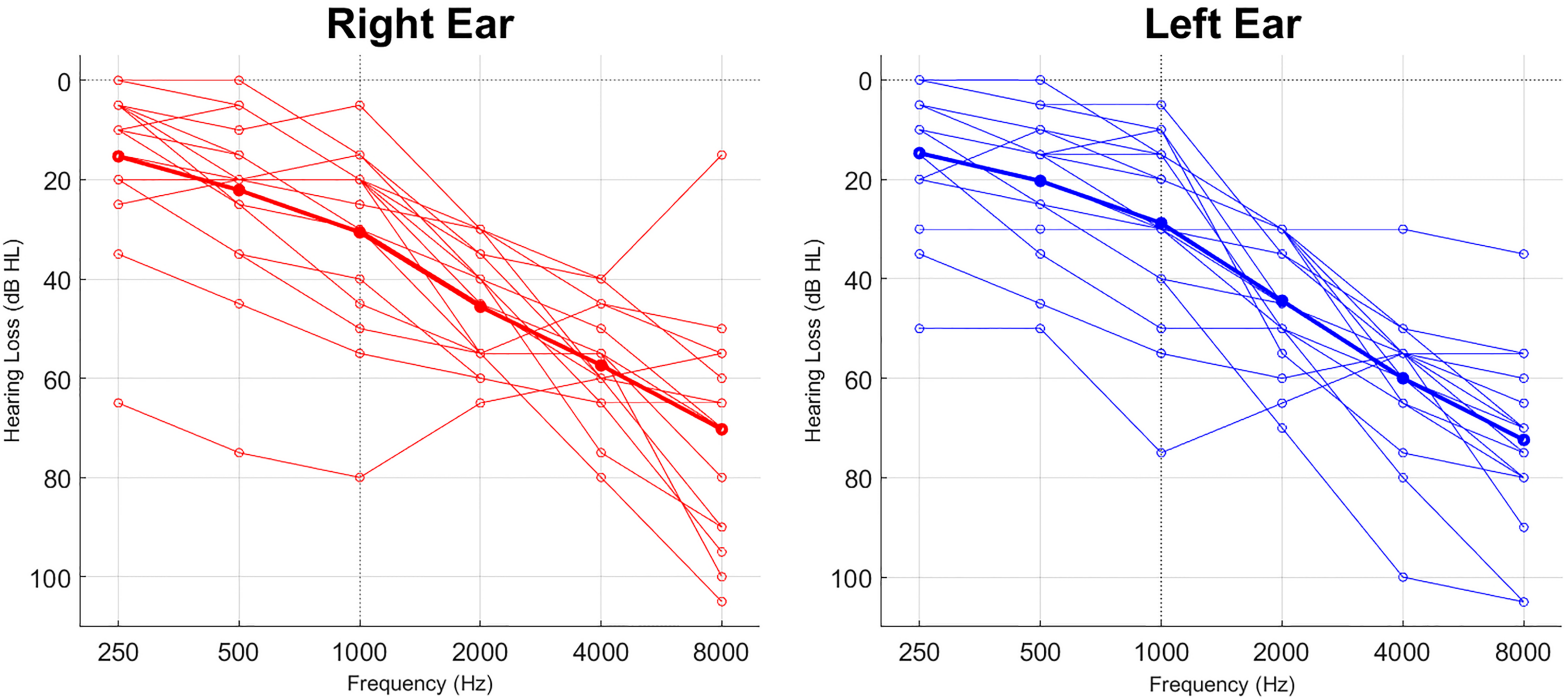
Audiograms of all the participants. The bold lines represent average audiograms across all the participants.

Seventeen Danish speaking experienced hearing aid users completed the study (14 males, 3 females) with a median age of 74 years (range: 56–79 years) and median hearing aid experience of 9 years (range: 2–37 years). The participants had moderate-to-severe symmetrical bilateral sensorineural hearing loss (Figure 1). Additional inclusion criteria were: (1) smartphone user, (2) no audiological complications such as fluctuating hearing loss, tinnitus and/or hyperacusis, and (3) must be able to travel to the laboratory during the study period. The participants were recruited through an internal database of participants via phone or e-mail.

### Study design

The study consisted of two laboratory visits and a 17-day field trial. The laboratory part of the study took place at WS Audiology HQ in Lynge, Denmark. Data collection was done in March 2022 for the first eleven participants and in November 2022 for the remaining six. Each part of the experiment is described in detail below.

### Visit 1

During the first visit, a new audiogram was obtained if the one on file was older than one year. Participants were then fitted with Signia Pure 312 7X receiver-in-canal (RIC) hearing aids. The hearing aids were fitted to the participant's hearing loss using Connexx (version 9.6.5.182). Own-voice-detection training was performed (12). This enables the hearing aid to detect the hearing aid wearer's own voice and to process it differently from external sounds. For this study it was important to filter out situations where the hearing aid wearer is not speaking, as own voice is louder at the position of the hearing aids than other speakers, leading to a higher SNR. Receivers and ear-tips were selected based on the fitting software's recommendation. Two participants requested and obtained fine-tuning (gain in the upper frequencies reduced by 3 dB). Otherwise, the default feature settings were used, and no special programs were added. The hearing aids were paired with a Samsung Galaxy S20 FE Android 11 mobile phone on which an internally developed EMA app was pre-installed. The Signia app was installed on the research phone to allow for remote fine-tuning in case participants requested it during the acclimatization phase. However, participants were instructed not to use it outside of any potential remote fitting session to ensure that the hearing aids are coupled to the EMA app and data collection is not disrupted. Volume control was the only available sound modification function. The participants were given a detailed explanation about what is expected of them during the field trial, how to operate the EMA app and fill out the surveys, and how to use the phone if they were not an Android user. The participants filled out one test survey before leaving the laboratory to ensure that they understood the task. Further, the participants were provided with illustrated printed step-by-step instructions about how to fill out the surveys and troubleshoot the EMA app.

### Field trial

The field trial was designed to last at least 17 days, but the timing of the second visit varied between participants based on their availability. The first three days were an acclimatization period during which the participants had a chance to get used to the sound of the study hearing aids. During this period, the participants were not required to do any tasks other than wear the hearing aids. The participants were encouraged to contact the study lead during the acclimatization period to ask any questions related to hearing aids or the study in general. After the three days, the study lead called the participants to inform them that the study period was going to start and ask them to prepare the equipment by ensuring: (a) the mobile phone is fully charged, (b) hearing aids are functioning as expected, (c) batteries are replaced and (d) a connection between hearing aids and the EMA app can be established. The participants could enable the “Do not disturb” function in the app during time periods where they did not want to be disturbed. Further, the “Do not disturb” function was enabled during participants’ normal sleeping hours for the duration of the study—this time window was individually chosen by each participant to fit their regular sleeping routines.

Then, the 14-day EMA period started, where the participants were prompted to answer EMA surveys. The specific EMA questions and response alternatives that were analyzed for this article are outlined in Table 1. The background for the chosen questions and response alternatives is given in the next subsection. The study was planned with six random survey prompts per day, but due to a technical error the participants were prompted to complete a survey by an app notification on average nine random times per day. The minimum interval between an answered survey and the next prompt, i.e., the next notification with a request to answer a survey, was 15 min. Additionally, up to four prompts based on objective data from the hearing aids could be delivered when the hearing aids detected speech. Those were only issued if at least one hour had passed since the last speech-detection prompt was delivered. The criteria for speech-detection prompts were that during the last five minutes (a) “speech in quiet” and “speech in noise” sound classes were flagged ≥40% of the time and (b) the median sound pressure level was ≥30 dB. This second criterion should be fulfilled for any speech situation, but as the app requires a combined criteria of level and class, the median level was specified here. These criteria were evaluated every 15 min resulting on average in 3.0 (SD: 1.0) speech-detection prompts per day. In addition to the app-prompted surveys, the participants were encouraged to self-initiate surveys if they would like to report a certain acoustical environment.

**Table 1:** English translation of the EMA questions and response alternatives. The original questionnaire was in Danish. Questions for “Speech understanding” and “Listening effort” were adaptive questions if the response to “Listening situation” was 3–6.

Category	Question	Response alternatives
Hearing aid satisfaction	How satisfied are you with the sound of your hearing aids in this situation?	1. Completely satisfied 2. Very satisfied 3. Satisfied 4. More or less satisfied 5. Dissatisfied 6. Very dissatisfied
Listening situation	Whom or what are you listening to right now?	1. I am not actively listening to anyone /anything 2. Music 3. Radio / TV / E-book / speech 4. Conversation with one person 5. Conversation with several people 6. Phone 7. Other
Speech understanding	How much speech do you understand in this situation?	0 (Nothing) 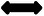 10 (Every word)
Listening effort	How much effort is required to listen to and understand sentences?	1. No effort 2. Very little effort 3. Little effort 4. Moderate effort 5. High effort 6. Very high effort 7. Extremely high effort


The prompts, meaning the requests to answer a survey, were delivered via an audible notification sound in the hearing aids and a visible notification on the screen of the smartphone. Once a prompt was issued, the notification was visible on the phone screen for 15 min before it disappeared if not answered. The EMA survey timed out 30 min after it was initiated if not completed. Prompts other than speech detection prompts were independent of the Bluetooth connection between hearing aids and the smart phone, but the survey could only be answered if there was a Bluetooth connection, to ensure the acquisition of the corresponding objective data. The objective hearing aid data that were collected are outlined in Table 2. Under ideal conditions objective data would have been collected from both hearing aids every 2.5 s. However, in reality, transmission is often not optimal resulting in a lower sampling rate. When pooled over all participants, the median sampling period was 2.52 s (75th percentile: 9.96 s, 90th percentile: 61.30 s). The hearing aid data collection began (a) 3 min before a prompt was issued, (b) when a user-initiated survey was started, and (c) every 15 min. It then continued until the connection was lost because hearing aids were not in the vicinity of the phone. Connection was indicated in the app by a connection sign and participants were instructed to restart the phone if they realized this sign was missing.

**Table 2:** Description of the logged hearing aid parameters that were analyzed for this study. The parameters which were logged and analyzed constitute a small subset of the environmental estimators operating in the hearing aid.

Parameter	Explanation
SNR estimate	Hearing aid estimate of the SNR before signal processing. This is only interpretable if speech is present.
Own voice detection	The hearing aid detection of whether the hearing aid user is currently speaking.
Acoustic class	The hearing aid classifies the acoustic situation into speech in quiet, speech in noise, noise, music, car, or quiet.

The study lead could monitor online how many surveys have been answered and when the last Bluetooth connection was established and could follow up with participants if any problems with data collection or participant compliance were detected. Hearing aid usage throughout the trial period was confirmed from the collected data after each participant completed the study.

### Choice of attributes and rating scales

There are no existing audiological rating scales that have been validated for use in EMA, though some studies looked into construct validity (13, 14) or showed that ratings change in the expected way for different SNRs (2). In previous EMA studies, ceiling effects or skewness towards positive ratings have been seen for several different attributes. For example, in a study by Schinkel-Bielefeld (6) the skewness for the distribution of ratings was 0.73 for satisfaction, 1.84 for speech understanding, 0.52 for listening effort and 2.4 for localization. While the exact skewness differs with attribute, it seems to be present in all the attributes. Here we focus on hearing aid satisfaction, speech understanding and listening effort as these are the attributes that are expected to depend on the SNR and are central to evaluating hearing aids.

For hearing aid satisfaction, Kerner et al. (15) and Jenstad et al. (2) used the end points “very satisfied” and “very dissatisfied” which have been taken from IOI-HA (16). As both studies observed ceiling effects, Schinkel-Bielefeld and colleagues (8, 17) added “absolutely delighted” to a five point rating scale where the highest positive option was initially “very satisfied.” While this resulted in an asymmetric rating scale, it seemed unnecessary to add another negative label as “very dissatisfied” was already scarcely used. However, feedback from native Danish speakers during the preparation of the current study was that such an enthusiastic label would not feel natural to Danish participants. Hence it was changed to “completely satisfied”.

In previous EMA studies several different questions for speech understanding have been used. These comprised the quality of speech understanding (5) or the ability to follow a conversation (3). Several studies ask for the amount of words/speech understood either as a slider (2, 18) or as a percentage (14, 19). The speech understanding question used here has also been used in previous studies (8, 17). Ten-point sliders with end points “every word understood” and “no word understood” have been used by Jenstad et al. (2) who showed that speech understanding measured with this rating scale changed with SNR.

The question of listening effort is based on the ACALES scale which has been validated in German language in the laboratory (20). Here the unlabeled intermediate steps have been discarded. Also, the option “only noise” was removed as the question is only asked if the participant indicated that there is speech present. The same question has been used in EMA by v. Gablenz et al. (5).

The above considerations resulted in different numbers of response options for each attribute. However, it is not uncommon in EMA that some, but not all attributes have the same number of response options, as often each question is evaluated in isolation (e.g., 5, 19, 21, 22).

### Visit 2

Following the field trial, the ANL and a rating test with the EMA app at different SNRs (“SNR test”), were conducted. The ANL and SNR tests were done after the field trial to ensure that participants have gained a stable representation of the rating scale before they did the SNR test. After the test, the participants returned the study equipment.

### Speaker set-up and stimuli

**Figure 2:**
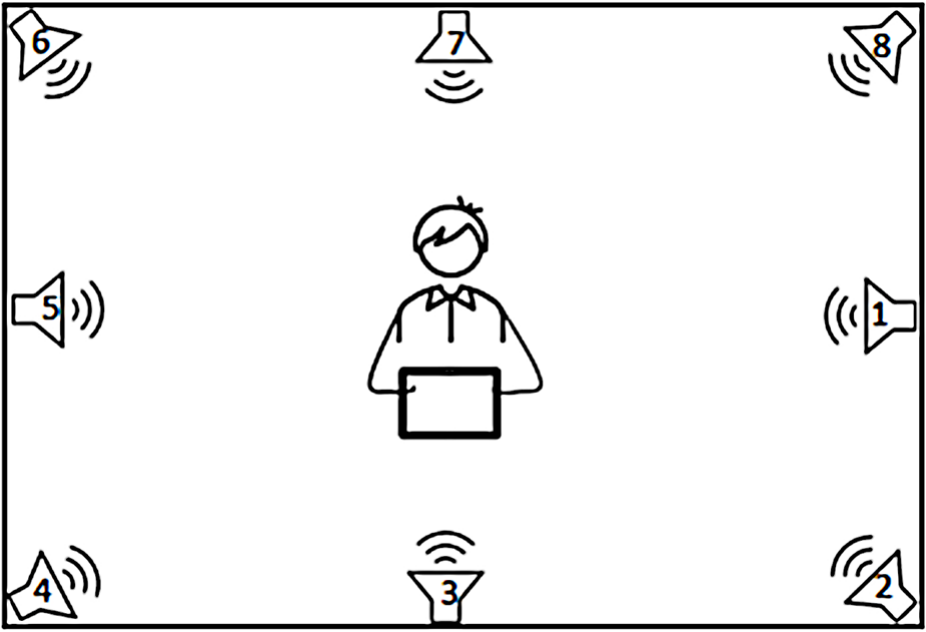
The laboratory speaker set-up.

All listening tests were performed in a highly absorbent listening room, isolated with 100 mm rock wool. The dimension of the room was 395 × 285 × 240 cm. Calculated reverberation time (RT60) was below 120 ms in the frequencies 0.125 to 8 kHz. In this room, a setup with eight loudspeakers was used (see Figure 2), where the speech was played from the front speaker located at 0-degree azimuth and noise was played from the remaining seven speakers. The participant was comfortably seated at the center of the loudspeaker ring.

Two different speakers (male and female) were used in two different noise signals (traffic and canteen noise). The speech materials were taken from audio books, i.e., (23) for male and (24, 25) for female speech. Different speech files were used for different ANL conditions and trials in the SNR Test. The speech files were 66 s long but were looped in case participants needed longer time. The sample rate/bit depth for speech and noise files was: 16 bit/44, 1 kHz. The canteen noise was a mono signal routed to different speakers with different delays between the eight speakers to avoid audible phase cancellation. Traffic noise was a stereo recording, with right and left channels routed to different speakers and temporal offsets applied. Canteen noise was stationary with peak energy at 433 Hz, while traffic noise was modulated with peak energy at 103 Hz. The same noise files were used in all trials for that noise type.

It has previously been shown that visual cues can significantly lower noise annoyance (26), hence, the noise was illustrated visually with a laminated printout (297 × 420 mm) depicting a busy canteen / traffic in front of the participant.

### The ANL test

The ANL test was administered via an iPad. The test started by measuring the most comfortable level (MCL). The initial speech signal was a female voice in quiet at 60 dB SPL. The participants were instructed to listen to a recording of the speaker telling a story, while adjusting the level of the speech signal in 5 dB steps using an up and down procedure: first, the participant increased the level of speech signal to where it was too loud to listen to, then they decreased the speech level until it became too soft to listen to. Finally, the participant adjusted the speech level to where it was the most comfortable to listen to and this was the measured MCL.

Then, the background noise level (BNL) was measured. The participants were instructed to listen to the same female voice telling a story, but this time, with background noise, starting with canteen noise. The participants first increased the background noise level in 5 dB steps to where they could no longer follow the narrator. We term this the high noise level (HNL). Then, they decreased the background noise level in 5 dB steps to where the speech became very clear and distinct. Finally, the participant adjusted the background noise level in 2 dB steps to where they would be willing to listen to the speech-in-noise for an extended period and this was the measured BNL.

Two BNLs were measured in the current study: speech level set to individual MCL measured in the first task, and speech level set to 65 dB SPL which corresponds to the level of a conversation in noise (27). The test with individual MCL may be more representative for media listening where hearing aid wearers can adjust the speech volume to their liking, while the test with fixed speech level is more representative for situations where there are fewer possibilities to adjust the volume of speech.

The ANL was calculated based on the individual MCL and BNL values (ANL = MCL–BNL) (28). As we were interested in SNRs that participants do not report / experience, we also calculated the “unable to follow” noise level (UNL) as MCL–HNL. A MATLAB script was used to conduct the test and calculate the ANL and UNL values. In total, four individual ANL and UNL values were acquired during the laboratory experiment: speech in canteen noise and speech in traffic noise × 2 (level of speech at MCL and at 65 dB SPL).

### The SNR test

The participants were asked to rate their satisfaction with hearing aids, speech understanding and listening effort while listening to speech-in-noise at different SNRs. They used the EMA app with the same questions and response alternatives for the three attributes as done during the field trial (see Table 1). It should be noted that the SNRs presented in the laboratory and the SNR estimates in the hearing aid do not match perfectly. Hence the hearing aid SNR estimates were also measured during the SNR test in the laboratory.

The speech signal was fixed at 65 dB SPL and the SNR varied between −10 and 20 dB SPL in 5 dB steps. Each of the four stimulus combinations (female/male talker in traffic/canteen noise) were presented seven times in random order. The participants indicated when they were done rating the sound file they were listening to by raising their hand, after which the study lead played the next sound file. Both the SNR test as well as the ANL test were performed with hearing aids on.

### Data analysis

As questions on speech understanding and listening effort were situation dependent, surveys were counted as completed if the first four questions enquiring about hearing aid satisfaction, listening environment, listening situation and background sounds were answered. Sampling rate for the objective data varied with stability of the connection. To get an assessment of the amount of collected objective data, we looked at each individual minute and counted all the minutes where at least one data point was collected. Unless otherwise noted, the SNR estimates from the hearing aids were analyzed only for situations that were classified as “Speech in Noise” or “Speech in Quiet”. Also, only situations where own voice of the hearing aid wearer was not detected were taken into account. The ANL test speech levels and background levels should follow a certain pattern, i.e., first high, then low and then in-between. Data from three participants deviated from this pattern by more than 3 dB in some of the trials. Hence it was assumed that instructions were not correctly followed and the complete data from these participants were discarded in all the analyses that included the ANL test.

For correlation analysis between real-life SNRs and ANL or UNL, it would be desirable to pool the results from the trials with different speakers and noise types to reduce potential noise in each single trial. To ensure that there were no significant differences between trials before pooling, two repeated measures one-way ANOVAs with speaker and noise type as independent variables were performed. Dependent variables were ANL and UNL, respectively.

Data analyses were mostly descriptive using boxplots or means with 95% confidence intervals. All error bars denote 95% confidence intervals obtained via bootstrapping with 50,000 repetitions. Bootstrapping was used as data distributions for some SNRs and ratings in the field trial were asymmetric. In cases where statistical inference tests were used, they are reported together with the *p*-value in the results section. All the statistical analyses were done in MATLAB 2019b.

As values of SNR estimates collected by the hearing aid during the laboratory tests did not exactly match the SNRs measured there, the hearing aid SNR estimates were mapped to the laboratory SNR values for determining the percentage of SNR estimates below the ANL and UNL.

## Results

### Acceptable noise level test

On a group level, no significant difference was found between ANLs with different background noise types and between the conditions where speech level was fixed and those where participants could choose their MCL (repeated measure one-way ANOVA, *p* = 0.71). Similarly, there was no significant difference between UNLs (repeated measures one-way ANOVA, *p* = 0.59). Collapsing across conditions, the group means for ANL and UNL were 11.0 dB SNR (SD: 5.8 dB SNR) and 2.2 dB SNR (SD: 6.2 dB SNR), respectively. To get a sense of the consistency of the ANLs and UNLs, mean absolute differences between measures for traffic and canteen noise were computed and averaged across participants. In addition, Pearson correlation analyses between the measures were done. Generally, the mean absolute difference was below 5 dB and all the compared measures except the UNL for individual MCLs (*p* = 0.07) were significantly correlated (*p* < 0.01). ANLs for individual conditions as well as MCLs for each participant can be found in the Supplementary Figure S1, Supplementary Table S1.

### SNR test in the laboratory

**Figure 3:**
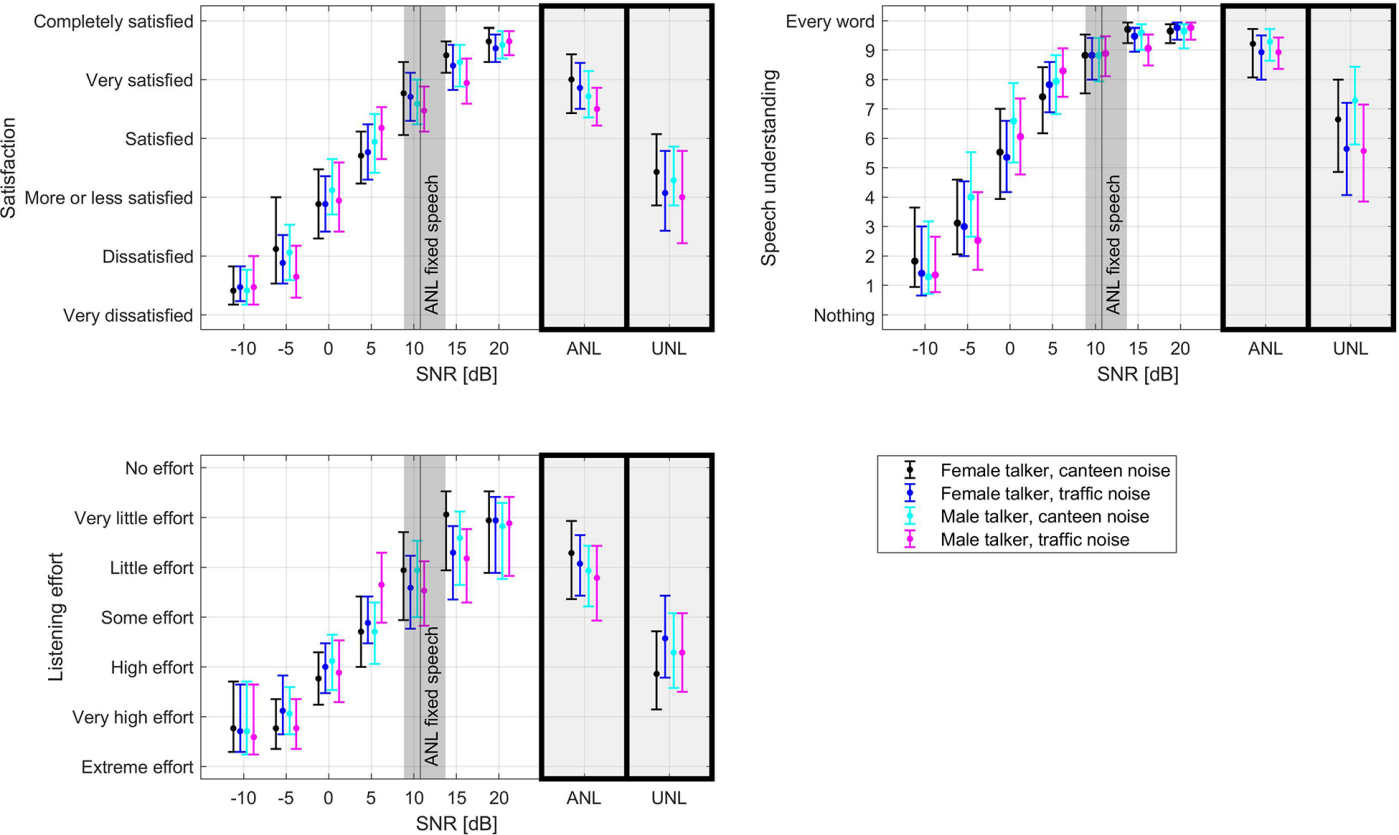
Average hearing aid satisfaction, speech understanding and listening effort ratings in the SNR test for different speech and background signals. Error bars denote 95% confidence intervals. The black line denotes the mean ANL captured with the ANL test with fixed speech level as this is identical to the speech level in the SNR test. The gray area around this line denotes the 95% confidence interval of the mean ANL. The boxes to the right of each plot contain the average ratings for the SNR value closest to the individual ANL and UNL.

SNRs presented during the SNR test in the laboratory ranged down to −10 dB SPL, which is 21.0 dB SPL below the average ANL of participants. Ratings for hearing aid satisfaction, speech understanding and listening effort spanned the entire rating scale (see Figure 3). Ratings for SNRs closest to the ANLs were rather positive, especially for speech understanding and satisfaction. Ratings at the UNLs were close to the response option “More or less satisfied” for hearing aid satisfaction and depending on the speaker and type of background noise between 5.6 and 7.2 for speech understanding. Listening effort for the UNL ranged mostly between “some effort” and “high effort” with the female speaker in canteen noise requiring slightly more than “high effort”.

### EMA field trial

#### Collected data

On average, participants answered 164 surveys (range: 29–273) of which 98 (range: 22–194), that is 60%, were reported to be in speech situations and hence contained questions on speech understanding and listening effort. The median survey completion time for each participant was on average 39 s (range: 20–100 s).

Collected objective data points per participants were on average 47,482 (range: 13,493–165,025). On average 83.4 h (range: 31.9–147.4 h) of objective data were collected per participant (counting each minute where at least one data point was collected as described in the methods section). For the full duration of the study the average total hearing aid wearing time was 247.3 h (range: 174.8–342.1 h). This includes the acclimatization time, except for one participant for whom no data were collected during the acclimatization time due to a technical error. Daily wearing time was on average 13.6 h (range: 7.1–16.3 h). Speech was detected by the hearing aid on average 27% (SD: 12%) of the wearing time.

**Figure 4:**
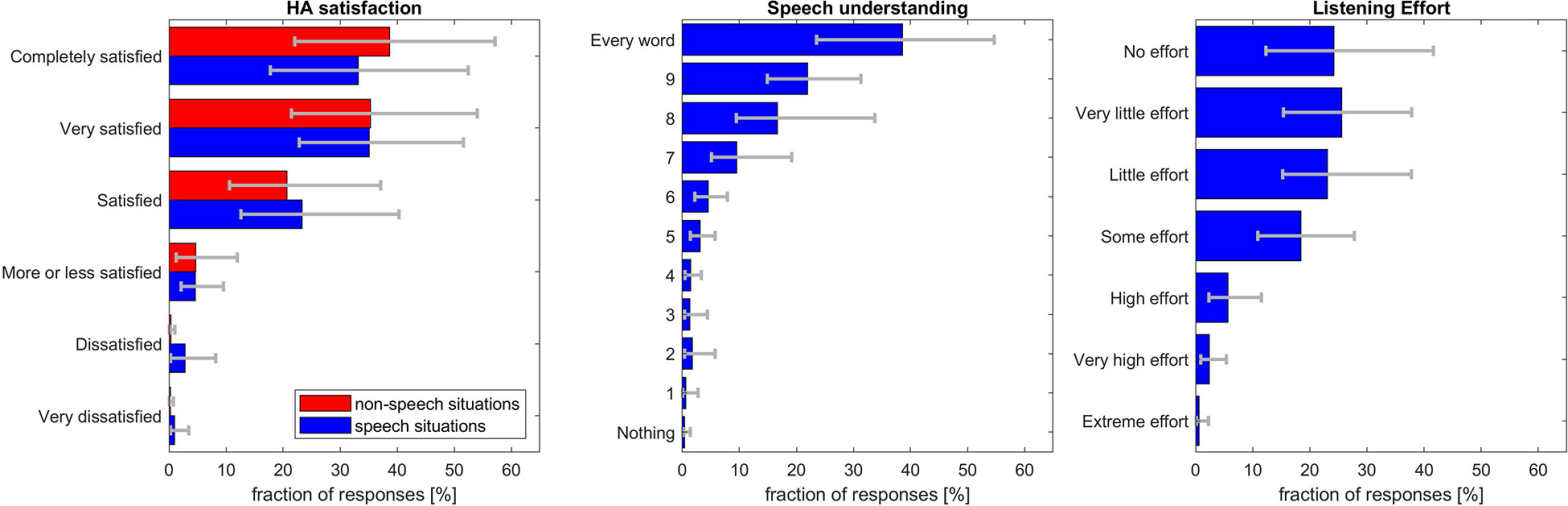
Ratings in the EMA field trial. Error bars denote 95% confidence levels. Note that speech understanding and listening effort were only rated if participants indicated that the listening situation contained speech.

The EMA ratings for hearing aid satisfaction, speech understanding and listening effort are shown in Figure 4. Most of the hearing aid satisfaction ratings were “satisfied” to “completely satisfied” (i.e. 91.6% (SD: 13.1%) in situations reported to contain speech, 94.7% (SD 10.2%) in non-speech situations). On average, 60.6% (SD: 37.1%) of the ratings for speech understanding were 9 or 10 (every word understood) and 91.3% (SD: 16.6%) were at 6 or above. Most of the listening effort ratings [72.9% (SD: 27.0%)] were “little” to “no effort”. Skewness was 0.68 for HA satisfaction in speech situations and 0.78 for HA satisfaction in non-speech situations, 1.79 for speech understanding and 0.40 for listening effort. To assess whether reactivity could have taken place, we compared the first week's ratings to those of the second week. No significant differences were observed between the two weeks (two-sided paired sample *t*-test, *p* > 0.2 for all three attributes).

#### Comparison to laboratory ratings

**Figure 5:**
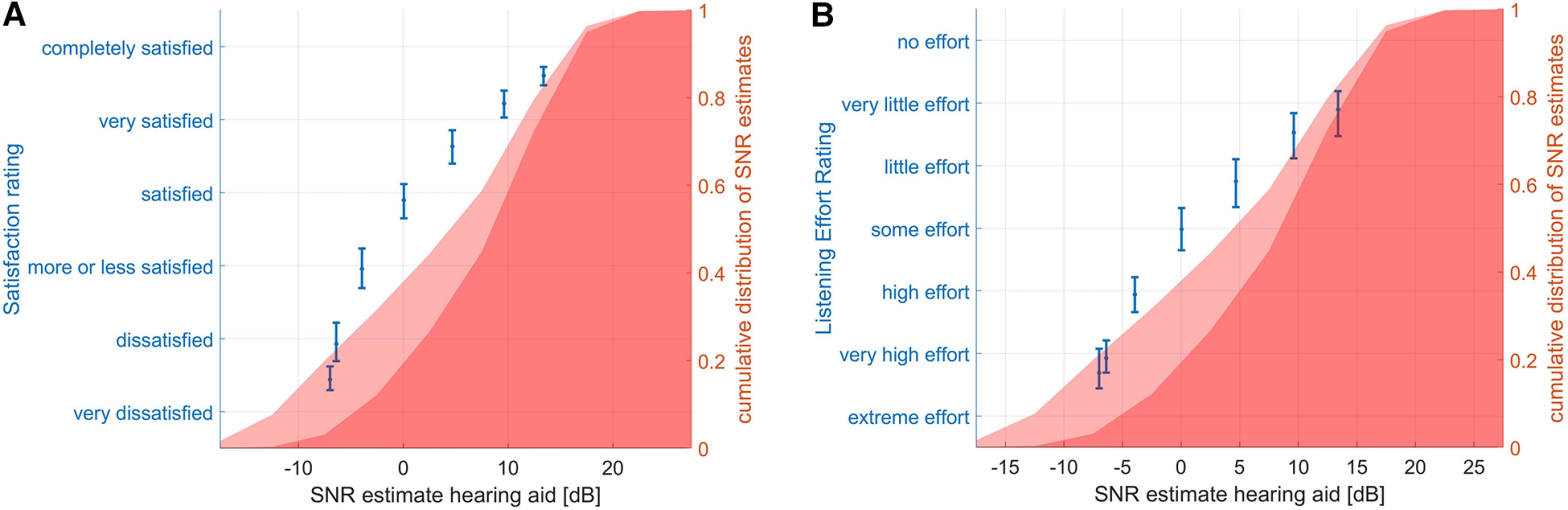
Cumulative distribution of SNR estimates during the field trial and ratings during the SNR test. The dark red area shows the SNR estimates for situations classified as speech. The lighter red area shows the SNR estimates during situations classified as speech, noise, or music. This serves as an upper limit for the prevalence of this SNR in case of misclassification of the situation as noise instead of speech in noise for negative SNRs. The error bars show mean and 95% confidence intervals of satisfaction (left plot) and listening effort (right plot) EMA ratings. Note that the distribution of SNRs is identical in (**A**) and (**B**) but was replicated for easier visual comparison to the ratings in the SNR test.

We analyzed how commonly the SNRs rated poorly (i.e., below the option “More or less satisfied” for satisfaction or in the lower half of the rating scale for speech understanding or listening effort) in the laboratory (Figure 3) occur in real life. Therefore, the corresponding ratings from the SNR test were plotted on top of a histogram of all the SNR estimates experienced in real life (see Figure 5). As the situations presented in the laboratory are only comparable to real-life speech situations, only the SNRs including speech (but no own voice detected) were considered. Less than 10% of situations during the field trial were in the SNR range that has been rated “more or less satisfied” or below on the satisfaction scale in the laboratory (Figure 5A). However, especially when negative SNRs are detected, the speech detection may confuse speech in noise with noise or even music situations. Hence, the 10% should be seen as a lower estimate. Considering also the situations classified as noise and music would likely overestimate the real prevalence of those SNRs in speech situations but would give an upper estimate of their prevalence in case of misclassification of a situation as noise instead speech-in-noise (light red in Figure 5A). Similarly, there were only a few situations where more than “some effort” was reported (Figure 5B).

#### Relation between ANL and SNRs experienced during the field trial

Of the collected SNR data during the field trial, the median percentage below the mean UNL across all conditions was 13.1% (SD: 9.7%). Mean percentage below the ANL was 32.0% (SD: 18.6%), see also Supplementary Figure S2. It should be noted that ANL and UNL are, for a given stimulus, essentially acceptable and “unable to follow” SNRs.

We conducted correlation analysis between individual ANLs and the real-life SNR estimates to assess whether there is a relationship between the two measures. It could be that there are only few situations with SNRs below ANL or UNL because those situations simply do not occur in the participants' environment. However, if correlation exists, it could be indicative of each person having some control to shape his or her environment to their needs.

As the ANL for different types of noise and speech levels (fixed and individually chosen) did not differ on the group level, the four types of ANL were averaged for the correlation analysis. If the ANL is indicative of which SNRs are not experienced in real life, it should correlate with the lowest SNRs experienced in the field trial. Hence, the 5th percentile of the real-life SNR estimates was included in the correlation analysis. No significant correlation between experienced SNR and measured ANL was found (Pearson's *r* = 0.18, *p* = 0.54). When doing the same analysis with the UNL, there was a moderate correlation (*r* = 0.54, *p* = 0.05, Supplementary Figure S3).

## Discussion

The EMA ratings for different SNRs in the laboratory revealed that the entire rating scale was used for all three tested attributes: hearing aid satisfaction, speech understanding and listening effort. This indicates that ceiling effects observed in previous studies (2, 8) and in the field trial of the current study are likely not caused by reluctance to use the lower end of the scale, or a desire to please the experimenter. Compared to the data by Schinkel-Bielefeld (6) skewness here was slightly higher for HA satisfaction and slightly lower for listening effort. Higher values for HA satisfaction may be caused by the change from “absolutely delighted” to “completely satisfied”. A reason behind using the entire range of the scale in laboratory, in contrast to real life, could be the clear differences between varying SNRs making it apparent which SNRs are worse than others and as such requiring worse ratings. In other words, participants have a benchmark against which to give their ratings in a laboratory setting. In EMA field trials, ratings are often spaced several hours apart and no direct comparison is possible potentially leading to greater contraction bias (29, 30). Nevertheless, different distribution of ratings in the SNR test and the field trial may well be caused by different distributions of SNRs between the two settings.

Indeed, hearing aid data indicate that those SNRs that were rated in the lower range of the scale in the laboratory were rare during the field trial. Only a minority (32%) of SNRs during the field trial were below the ANL measured in the laboratory and even less (13%) below the UNL. At 11.0 dB SPL, the mean ANL measured here was slightly lower than previously measured ANLs in Danish normal hearing individuals [e.g., (31): 12.6–16.6 dB SPL] and in-between the previous results for part-time users and full-time hearing aid users in the US (32).

The SNRs in the laboratory test corresponding to ANLs were generally still rated very positively (Figure 4). Even the laboratory SNRs corresponding to UNLs, that is the SNRs where participants could no longer follow the narrator, were rated as “more or less satisfied” in terms of hearing aid satisfaction. Speech understanding was rated in the upper range of the scale and “high effort” was needed to understand at the UNLs. If the participant in the field trial had adapted or left such difficult listening situations before answering the EMA survey, then the lower part of the scale may not be relevant for ratings obtained in everyday life. This should be considered when designing new rating scales for EMA. As the concepts of speech understanding and listening effort are different but somewhat related, sometimes only one of the two is included in a study (33). The larger spread of responses for listening effort compared to speech understanding indicates that listening effort may be more suitable to show differences between two tested conditions than speech understanding.

There are fundamental differences between laboratory experiments and EMA. In laboratory experiments, participants can be asked to pay attention to speech signals that are incomprehensible and hence the very negative response alternatives can be used. In real life, participants would be very unlikely to endure such situations and then the very negative response options may not be relevant. As such, it may be appropriate to use different rating scales for EMA and laboratory experiments. It has previously been discussed that unipolar and bipolar scales substantially differ with respect to measurement properties (34). Bipolar scales are often used in real-life experiments (2, 8). However, it may be unintuitive to rate different levels of dissatisfaction in real life, but a unipolar scale assessing different levels of satisfaction could be more appropriate and lead to a larger range of scale being used. Although, skewness toward positive hearing aid satisfaction ratings in EMA is still likely even when using unipolar scales (7).

Our results showing a limited number of real-life situations with unfavorable SNRs during the field trial are in agreement with Smeds et al. (35) and Wu et al. (36) who also found mostly positive SNRs in real life. Based on the sound environment classification data collected over 13 months, Humes et al. (37) concluded that hearing aid wearers mostly choose favorable environments for hearing aid use with speech in noise at overall levels >75 dB occurring less than 5% of the time.

Smeds et al. (35) analyzed 75 recordings from different situations of 20 satisfied hearing aid wearers, computing the SNR from unweighted speech and background sound levels. The distribution of SNRs spanned a similar range as found here and comprised SNRs of −10 dB, as used in the SNR-test in the current work. SNRs from A-weighted speech and noise levels found by Smeds et al. (35) and Wu et al. (36) were higher. This is expected as A-weighting affects the low frequency components of the noise more than speech.

Looking at the SNR of different noise categories in Smeds et al. (35), only the median SNR of the noise category quiet, which comprised more than a third of the recorded situations exceeds the mean ANL measured here. Median SNRs for noise categories “car”, “public transport” and department store' are below the UNL measured here. The distribution of SNRs in Smeds et al. had a peak between 2 and 6 dB SNR and the percentage of their SNR distribution below the ANL and UNL in our study seems larger. Christensen et al. (38) analyzed in-market data collected from Oticon Opn hearing aids. The percentage of real-life SNRs in their work that is below the mean ANL and UNL in our study is larger than the percentages we found. However, their real-life SNRs were generally higher than the ones found here. While this comparison to existing literature seems to indicate that there might be a larger percentage of real-life SNRs below the ANL than measured in this study, it should be noted that this comparison is across different subject groups and hence is not necessarily valid.

The question is what the underlying reasons for the rare occurrence of unfavorable SNRs are. There are very few situations where humans have no control over the noise level (e.g., thunder storms). Some situations are controlled by the society (e.g., there are regulations for traffic noise) and some situations an individual has control over. Even if the noise cannot be changed, individuals have the choice to abort a conversation or continue it elsewhere. However, there may be consequential reasons for not doing this. For example, if work colleagues go to a very noisy canteen every day and it is important to the individual to spend time with them, one may decide to endure the noisy canteen from time to time.

The ANL by instruction indicates which SNRs participants are willing to listen to for a long time and the UNL at which SNRs they cannot follow the narrator anymore. Hence, presumably they would try to reduce the amount of listening situations at SNRs below the ANL and even more so below the UNL. Our hypothesis that the ability of individuals to adapt listening situations to their needs would lead to correlations between the ANL and lesser experienced low SNRs did not hold. However, there was a moderate correlation between the UNL and the 5th percentile of the experienced SNRs. This correlation to an extent supports the hypothesis that difficult situations may be avoided / modified. Avoidance or modification of difficult situations is in line with an EMA study by Schinkel-Bielefeld (6) showing that different acoustic environments are encountered depending on the hearing aid program used throughout the day and Borschke et al.^1^ showing varying modifications, acoustic environments and wearing behaviour depending on the hearing aid program used throughout the day. If individuals avoid or modify certain situations based on insufficient hearing aid performance, this has implications for EMA beyond the use of rating scales. In particular, hearing aid performance may be overestimated and sensitivity when comparing different hearing devices or programs may be reduced. When different conditions are compared, collection of objective data in addition to EMA can make differences in avoidance or modification behaviour discernable. Hence, whenever possible, the objective data should be collected in addition to subjective ratings when looking for these effects. Potentially, the objective data can even showcase adaptation of situations based on hearing aid performance without subjective ratings, and as such, could be an inobtrusive way to collect information about preferences of hearing aid features or programs.

It should also be noted that a potential driver of correlation between UNLs and real-life SNRs could be that the acoustic environments participants experience in everyday life shape their ANLs and UNLs. For example, if one spends most time in quiet environments, not necessarily due to purposeful avoidance because of discomfort / hearing difficulty, one's sensitivity to noise in turn may be higher. Similarly, if one spends a lot of time in noisy environments, one's sensitivity to noise may be lower. Whether it is the UNLs that guide the types of environments one spends time in, or whether it is one's everyday environments that determine their UNLs cannot be disentangled from the current data.

### Limitations of the study

As most EMA studies in audiology, this study was conducted with a small sample size and should be verified by a larger study.

Also, several limitations of the novel UNL measure should be noted. Originally this was just meant to be a reversal point in the ANL procedure determined with a larger step size than the final ANL value. However, communication to the participants did not treat it as any less important than the end point of the ANL test. Hence, presumably participants used the same care for both measurements. The novel UNL measure has not yet been validated. Here it is assumed that the SNR where one cannot follow a conversation is similar to the SNR where participants start changing their behavior to improve the SNR. This assumption needs testing. Additionally, a new measure directly addressing unacceptability of the SNR and avoidance / modification behavior may lead to greater correlations with real-life SNRs, though this would also require validation. The fact that three participants did not follow instructions suggests that the ANL would have benefitted from training.

Collection of the SNRs was dependent upon participants carrying the research phone with them and reconnection between hearing aids and research phone when hearing aids were within reach of the phone was not always successful. It could, however, be fixed by restarting the phone. For this reason, only about a third of the objective data were collected. However, technical reconnection problems were independent of the acoustic situation and should not affect the representativeness of the collected data. As participants may be less likely to interact with the research phone in social situations, there may be an underestimation of those situations (8). Also, difficult situations with high cognitive load could be a reason to skip a survey, especially if there is no possibility to report on the situation with some delay. While the exact prevalence of negative SNRs found in this study should be interpreted with care, this study still shows that SNRs corresponding to negative ratings are rare in everyday life.

Automatic estimation of SNRs by hearing aids is difficult. Even if the SNR is estimated accurately, there is no way of knowing whether the hearing aid wearer is paying attention to the estimated target signal (i.e., the prominent talker) or not. Further, SNR estimation accuracy decreases for low SNRs and as such low SNRs may be underestimated. The difficulty of a listening situation does not only depend on the SNR, but on many other factors. While a lower SNR can cause greater listening effort, a familiar person talking slowly with clear articulation about a known topic is easier to understand than an unfamiliar voice with a heavy accent talking rapidly about an unknown topic. All those influences were not captured in the current study.

## Conclusion

The results showed that the full rating scale was used in the laboratory, while the ratings in real life were strongly skewed towards the positive end of the response scales. Hence, reluctance to give negative feedback is likely not an underlying reason for the positive ratings in real life. The UNL, meaning the SNR where participants could not follow the speech anymore, was rated at the mid-point of the speech understanding scale and at “more or less satisfied” on the hearing aid satisfaction scale. This questions how relevant the lower part of the rating scales used in this study is for rating real-life situations. The hearing aid data collected during the EMA trial were related to participants' UNL. These data also indicated that people do not seem to frequently experience listening situations at SNRs where they are dissatisfied with their hearing aids. This could be the reason for the overly positive hearing aid satisfaction ratings in EMA studies. It remains unclear to what extent the scarcity of such situations is due lack of encounters or adaptation of the acoustic environment in dissatisfactory situations.

## Data Availability

The raw data supporting the conclusions of this article will be made available by the authors, without undue reservation.

## Footnotes

^1^Borschke I, Jürgens T, Schinkel-Bielefeld N. How individuals shape their acoustic environment—implications for hearing aid comparison in ecological momentary assessment. Manuscript submitted for publication (2022).
