# Supplementary material for: Reasons for ceiling ratings in real-life evaluations of hearing aids: the relationship between SNR and hearing aid ratings
Source: Front Digit Health. 2023 Aug 3;5:1134490. doi: 10.3389/fdgth.2023.1134490 (PMC10436089; doi:10.3389/fdgth.2023.1134490)
Supplement: Supplementary file 1 [file Datasheet1.docx]

Supplementary Material

Reasons for ceiling ratings in real-life evaluations of hearing aids: The relationship between SNR and hearing aid ratings

Nadja Schinkel-Bielefeld*, Jana Ritslev, Dina Lelic

*** Correspondence:** Corresponding Author: nadja.schinkel-bielefeld@wsa.com

# Supplementary Figures and Tables

| Participant | 1 | 2 | 3 | 4 | 5 | 6 | 7 | 8 | 9 | 10 | 11 | 12 | 13 | 14 |
| --- | --- | --- | --- | --- | --- | --- | --- | --- | --- | --- | --- | --- | --- | --- |
| MCL Canteen [dB SPL] | 59 | 51 | 57 | 55 | 51 | 69 | 66 | 52 | 64 | 68 | 64 | 62 | 61 | 64 |
| MCL Traffic [dB SPL] | 60 | 46 | 60 | 50 | 49 | 68 | 66 | 54 | 58 | 70 | 62 | 59 | 62 | 60 |

**Table S1: Individual MCL levels for all participants that followed the instructions in the ANL test correctly.**


a)
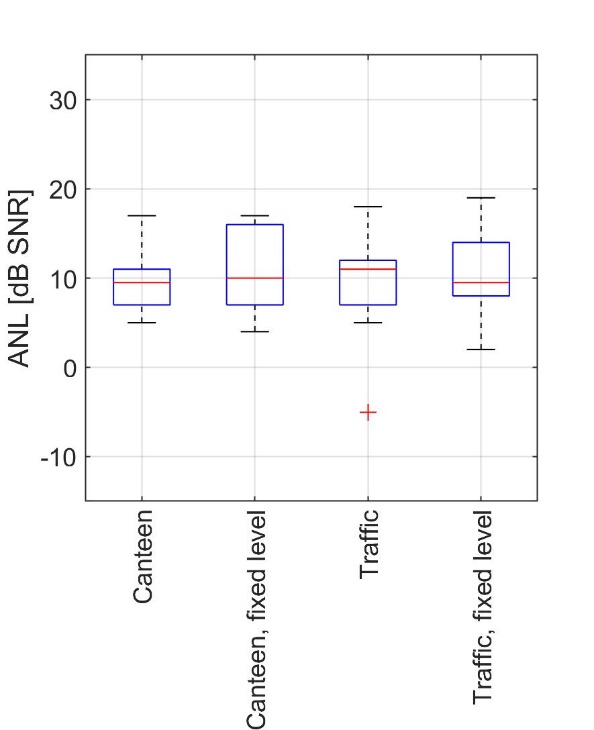
 b)
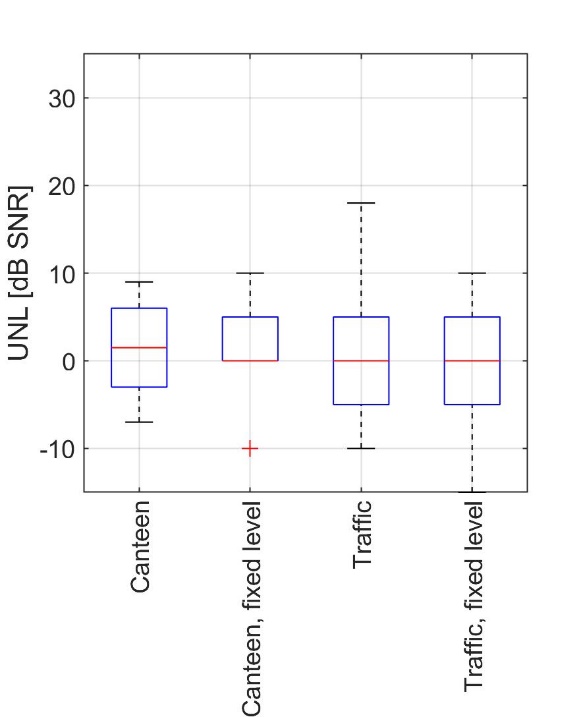


Figure S1: a) ANL and b) UNL for both types of noise and for speech levels at individual MCL and fixed to 65 dB SPL.


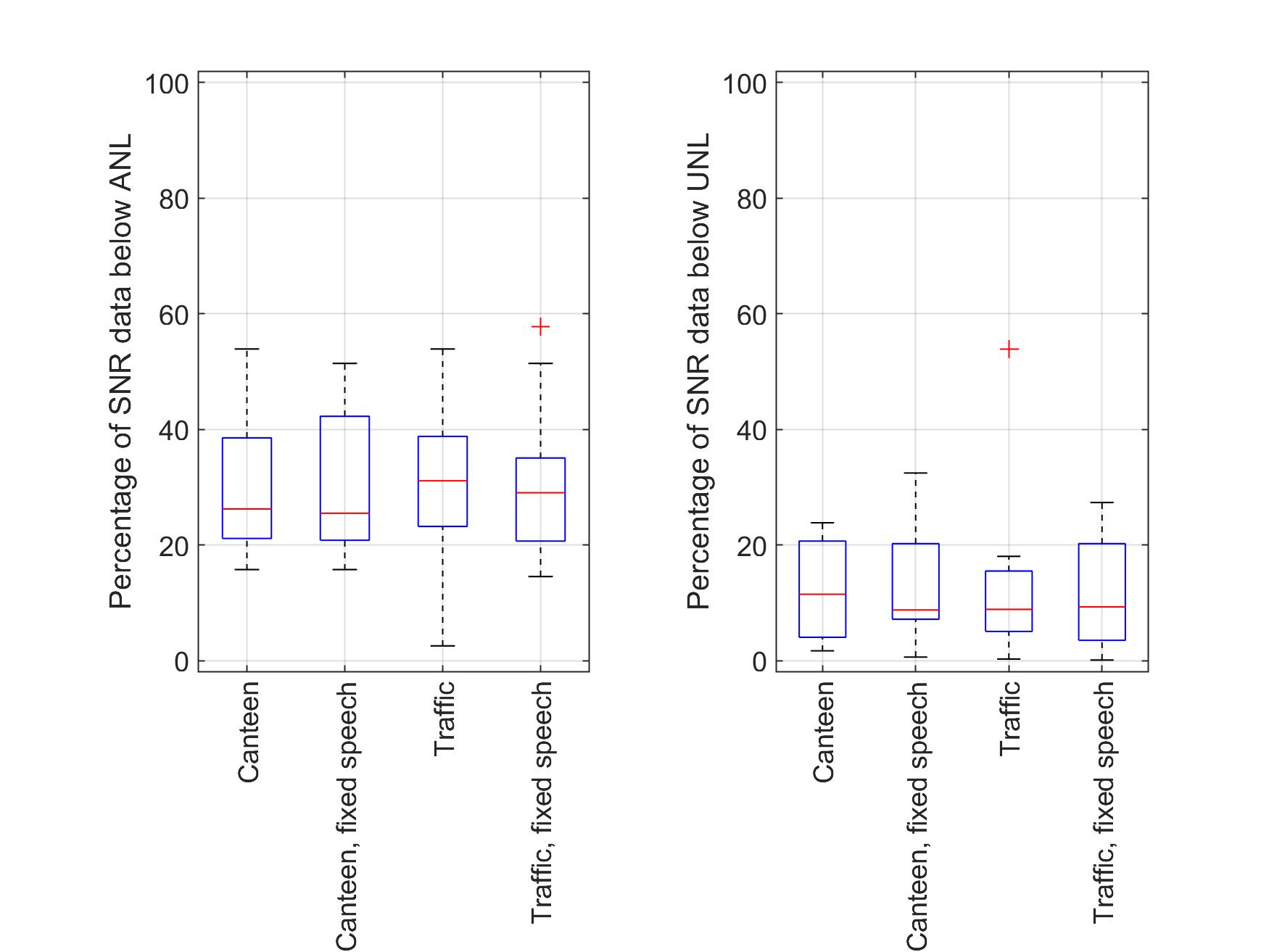


Figure S2: Percentage of the SNR estimates collected during the field trial that was lower than the acceptable noise level (left plot) or the unacceptable noise level (right plot).

1.
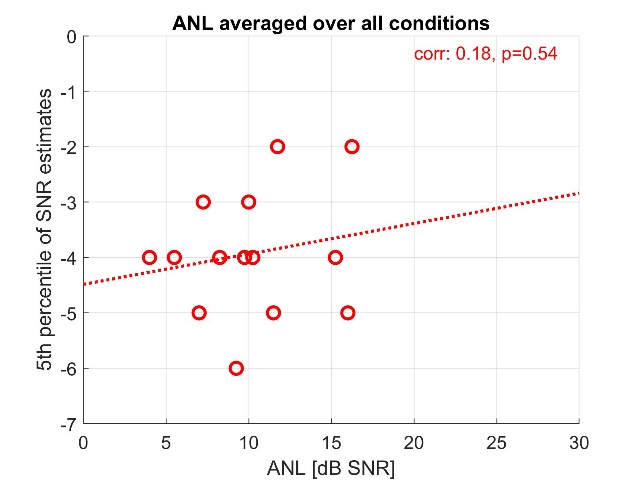
 b)
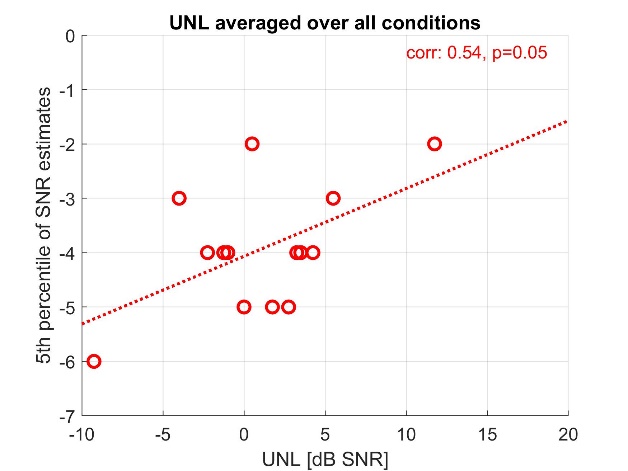


Figure S3: Correlations between 5^th^ percentile of experienced SNRs and ANL (left) and between 5^th^ percentile of experienced SNRs and UNL (right). The red dotted line represents the best linear fit to the data.
